# Meta-analysis of the characteristic expression of circulating microRNA in type 2 diabetes mellitus with acute ischemic cerebrovascular disease

**DOI:** 10.3389/fendo.2023.1129860

**Published:** 2023-02-14

**Authors:** Feifei Hu, Lei Liu, Zhijian Liu, Mingfeng Cao, Guanghong Li, Xinhuan Zhang

**Affiliations:** ^1^Department of Endocrinology, The Second Affiliated Hospital of Shandong First Medical University, Taian, Shandong, China; ^2^The Second Affiliated Hospital of Shandong First Medical University, Taian, Shandong, China

**Keywords:** microRNA, type 2 diabetes mellitus, acute ischemic cerebrovascular disease, circulating, meta-analysis

## Abstract

**Objective:**

To comprehensively evaluate the characteristics of the circulating microRNA expression profile in type 2 diabetic patients with acute ischemic cerebrovascular disease by systematic evaluation and meta-analysis.

**Methods:**

The literatures up to March 2022 related to circulating microRNA and acute ischemic cerebrovascular disease in type 2 diabetes mellitus were searched and screened from multiple databases. The NOS quality assessment scale was used to evaluate methodological quality. Heterogeneity tests and statistical analyses of all data were performed by Stata 16.0. The differences in microRNA levels between groups were illustrated by the standardized mean difference (SMD) and 95% confidence interval (95% CI).

**Results:**

A total of 49 studies on 12 circulating miRNAs were included in this study, including 486 cases of type 2 diabetes complicated with acute ischemic cerebrovascular disease and 855 controls. Compared with the control group (T2DM group), miR-200a, miR-144, and miR-503 were upregulated and positively correlated with acute ischemic cerebrovascular disease in type 2 diabetes mellitus patients. Their comprehensive SMD and 95% CI were 2.71 (1.64~3.77), 5.77 (4.28~7.26) and 0.73 (0.27~1.19), respectively. MiR-126 was downregulated and negatively correlated with acute ischemic cerebrovascular disease in type 2 diabetes mellitus patients, its comprehensive SMD and 95% CI were -3.64 (-5.56~-1.72).

**Conclusion:**

In type 2 diabetes mellitus patients with acute ischemic cerebrovascular disease, the expression of serum miR-200a, miR-503, plasma and platelet miR-144 was upregulated and the expression of serum miR-126 was downregulated. It may have diagnostic value in the early identification of type 2 diabetes mellitus with acute ischemic cerebrovascular disease.

## 1 Introduction

Type 2 diabetes mellitus (T2DM), a metabolic disease related to the increased risk of central nervous system diseases, has attracted increasing attention because it can lead to electrophysiological, structural, neurochemical and degenerative changes in the nervous system and seriously damage neural function (1, 2). Studies have shown that T2DM increases the risk of ischemic stroke by 3-4 times (3). The prognosis of T2DM patients with ischemic stroke is poor, and the mortality is high. Therefore, it is important to improve the prognosis of patients by increasing the early diagnosis rate of T2DM with acute ischemic cerebrovascular disease (AICVD) and taking effective intervention measures in time. At present, the methods used to diagnose T2DM with AICVD are limited, mainly including magnetic resonance imaging (MRI) and CT angiography (CTA). However, the implementation of these two examinations has certain limitations and contraindications for individual patients. Therefore, we hope to find an examination method that is easy to operate, has no side effects, is widely applicable and has objective diagnostic indicators as an effective means to identify AICVD early in T2DM.

MicroRNAs (miRNAs, miRs), small noncoding RNAs, are important regulators of many biological processes, such as cell growth, apoptosis, cell proliferation, embryonic development and tissue differentiation (4). They play key roles in mediating the physiological and pathogenic functions of diabetes and diabetic complications (5, 6). Many studies have shown that miRNAs can be used as a sensitive biomarker of secondary brain injury (7). In recent years, an increasing number of studies have shown that the specific expression of some miRNAs in blood plays an important role in the pathogenesis of AICVD in T2DM. Unfortunately, at present, there are few clinical studies on circulating miRNA and AICVD in T2DM, and the conclusions are still inconsistent. The characteristics of its expression profile in T2DM with AICVD have not been accurately evaluated.

The purpose of this study is to further clarify the characteristics of the circulating microRNA expression profile in T2DM with AICVD by reviewing the research results of literature, and in addition to provide evidence for circulating microRNA with potential diagnostic value in T2DM with AICVD.

## 2 Methods

### 2.1 Literature retrieval strategy

The PubMed, Embase, Cochrane Library, CNKI, Wanfang and Vip databases were searched to obtain relevant literature published up to March 2022. We used the keywords and subjects both in Chinese and English as follows: “diabetes mellitus or diabetes or T2DM”; “miRNA or microRNA”; “cerebrovascular disease or stroke or acute or cerebral infarction or cerebral ischemia”.

### 2.2 Selection criteria

The inclusion criteria were as follows: (1) the study must have the miRNA expression profile of a human model of T2DM with AICVD; (2) the patients with T2DM complicated with AICVD were the experimental group, and the healthy group, the patients with T2DM alone and the patients with AICVD alone were the control group. The general information of these groups, such as sex and age, was balanced and comparable; (3) the relative miRNA expression must be reported by miRNA microarray or qRT PCR; the miRNA detection methods and reagent sources in the literature were clear; (4) the sample size and source of the experimental group and the control group were clear; (5) the mean and standard deviation of miRNA expression in the experimental group and the control group, or the relevant data that could be used to calculate the above indicators, could be obtained; and (6) all patients with T2DM met the WHO diagnostic criteria, and all patients with AICVD were diagnosed by brain MRI. The exclusion criteria were as follows: (1) not meeting the inclusion criteria; (2) simple descriptive literature without a control group; (3) animal research and cell research; (4) overview, case report, abstract collection, meeting and letter; (5) repetitive literature; and (6) literature for which relevant data could not be obtained.

### 2.3 Data extraction and management

According to the inclusion and exclusion criteria, we strictly screened the literature and extracted the following data: the first author, the year of publication, the country of study, the source of samples, the list of up- and downregulated miRNAs, miRNA detection methods, the sample size, mean and standard deviation of the experimental group and the control group. If the mean and standard deviation cannot be obtained directly, we will obtain them through calculation. If the original data cannot be obtained directly, we will contact the author to obtain them. If different types of AICVD or different sample sources were mentioned in the same document, data extraction and corresponding analysis were carried out respectly.

### 2.4 Quality assessment

All included studies were case−control studies, so the Newcastle−Ottawa Scale (NOS) quality evaluation scale was used to assess the quality of the included studies. The scale includes three aspects: the selection method of the case group and control group, the comparability of the case group and control group, and the exposure evaluation method. The scoring range is 0~9 points, in which 5~9 points are high quality and 0~4 points are low quality.

### 2.5 Statistical analysis

Stata 16.0 was used to statistically analyze the expression of each miRNA. The heterogeneity of this study was tested by the Q test and I^2^ test: if I^2^<50%, a fixed effect model was used, and if I^2^>50%, a random effect model was used. If the heterogeneity was still large, subgroup analysis and regression analysis were conducted to analyze the source of heterogeneity. The stability and reliability of the selected studies were discussed through sensitivity analysis. Finally, publication bias was evaluated by funnel chart and Egger’s test.

## 3 Results

### 3.1 Included studies and their characteristics

**Figure 1:**
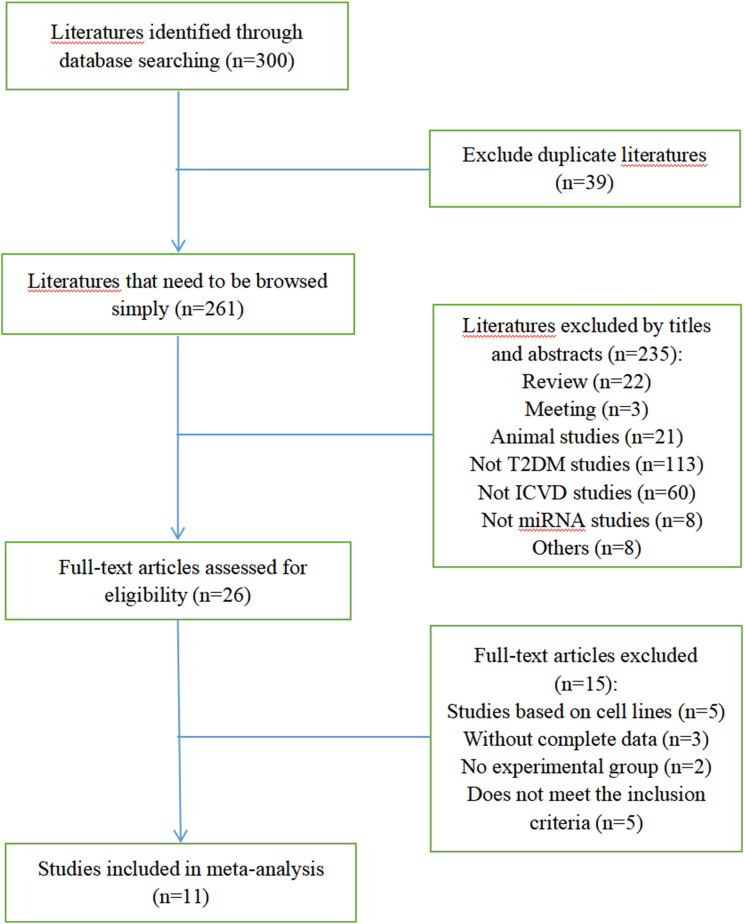
Literature screening flow chart.

**Figure 1** shows the selection process of the studies. There were 300 studies relevant to the search strategy, of which 39 were duplicates. A further 235 of these articles were excluded by browsing the titles and abstracts. After evaluating the full texts of the remaining studies, another 15 articles were excluded. Finally, 11 articles containing 486 cases and 855 controls were included in this meta-analysis (8–18). Details of the included studies are shown in **Table S1**.

### 3.2 Quality evaluation results

The NOS quality evaluation scale was used to assess the quality of 11 studies, as shown in **Table 1**. The total NOS score of all studies was between 6-9, and all the included studies were considered to be of high quality.

**Table 1:** Quality evaluation of 11 included documents.

Study	Selection	Comparability	Exposure	Scores	Source of case samples	Number of cases
Fan Cai 2018	✮✮✮	✮✮	✮✮✮	8	Shanghai Ruijin Hospital Affiliated to Shanghai Jiao Tong University School of Medicine	15
Saba Sheikhbahaei 2019	✮✮✮✮	✮✮	✮✮✮	9	Alzahra hospital in Isfahan, Iran	15
Xiaqing Guo 2016	✮✮✮	✮	✮✮	6	Department of Neurology, Huaihe Hospital, Henan University	74
Zhen Liu 2020	✮✮✮	✮✮	✮✮	7	Guangyuan Traditional Chinese Medicine Hospital	132
Dila Na 2021	✮✮✮	✮✮	✮✮✮	8	The Seventh Affiliated Hospital of Xinjiang Medical University	92
Yuming Long 2015	✮✮✮	✮✮	✮✮✮	8	Xiangya Hospital, Central South University	12
Kaishun Meng 2019	✮✮✮	✮✮	✮✮	7	Endocrine Department of Sanya People’s Hospital	27
Xiaomei Duan 2014	✮✮✮	✮✮	✮✮✮	8	Xiangya Hospital	6
Yuefu Jiang 2021	✮✮✮	✮	✮✮✮	7	Affiliated Hospital of Southwest Medical University	15
Shuisheng Yang 2015	✮✮✮	✮✮	✮✮✮	8	Department of Endocrinology, Xiangya Hospital	58
Mauro Giordano 2020	✮✮	✮✮	✮✮	6	the Hospital of Marcianise, ASL Caserta, Italy	21/19

NOS uses the semi-quantitative principle of star system to evaluate the quality of literature, and the number of stars represents the score.

### 3.3 Results of the meta-analysis of the expression of each circulating miRNA in T2DM patients with AICVD

#### 3.3.1 Analysis results of circulating miR-223

**Figure 2:**
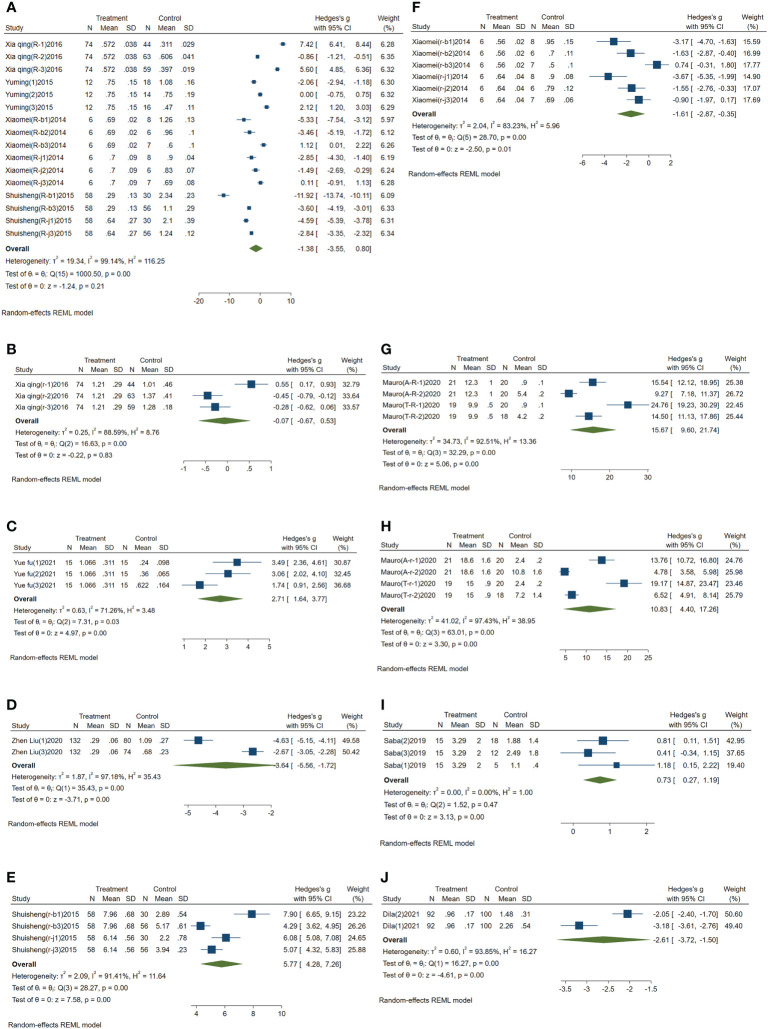
Forest plot of circulating miRNAs in type 2 diabetes mellitus with acute ischemic cerebrovascular disease. **(A)** Forest plot of circulating miR-223. **(B)** Forest plot of circulating miR-210. **(C)** Forest plot of circulating miR-200a. **(D)** Forest plot of circulating miR-126. **(E)** Forest plot of circulating miR-144. **(F)** Forest plot of circulating miR-146a. **(G)** Forest plot of circulating miR-195-5p. **(H)** Forest plot of circulating miR-451a. **(I)** Forest plot of circulating miR-503. **(J)** Forest plot of circulating miR-708-5p.

Meta-analysis results of circulating miR-223: I^2^ was 99.14%. Because I^2^ was greater than 50%, the comprehensive SMD and 95% CI obtained by using the random effect model was -1.38 (-3.55~0.80), P>0.05, which was not statistically significant, as shown in **Figure 2A**.

#### 3.3.2 Analysis results of circulating miR-210

Meta-analysis results of the circulating miR-210: I^2^ was 88.59%, and the comprehensive SMD and 95% CI obtained by using random effect model was -0.07 (-0.67~0.53), P>0.05, which was not statistically significant, as shown in **Figure 2B**.

#### 3.3.3 Analysis results of circulating miR-200a

Meta-analysis results of circulating miR-200a: I^2^ was 71.26%. The comprehensive SMD and 95% CI obtained by using the random effect model was 2.71 (1.64~3.77), P<0.05, which was statistically significant. The expression of miR-200a in the experimental group was upregulated compared with that in the control group, as shown in **Figure 2C**.

#### 3.3.4 Analysis results of circulating miR-126

Meta-analysis results of circulating miR-126: I^2^ was 97.18%. The random effect model was used to obtain the comprehensive SMD, and the 95% CI was -3.64 (-5.56~-1.72), P<0.05, which was statistically significant. The expression of miR-126 in the experimental group was downregulated compared with that in the control group, as shown in **Figure 2D**.

#### 3.3.5 Analysis results of circulating miR-144

Meta-analysis results of circulating miR-144: I^2^ was 91.41%. The comprehensive SMD and 95% CI obtained by using the random effect model was 5.77 (4.28~7.26), P<0.05, which was statistically significant. The expression of miR-144 in the experimental group was upregulated compared with that in the control group, as shown in **Figure 2E**.

#### 3.3.6 Analysis results of circulating miR-146a

Meta-analysis results of circulating miR-146a: I^2^ was 83.23%. The random effect model was used to obtain its comprehensive SMD, and the 95% CI was -1.61 (-2.87~-0.35), P<0.05, which was statistically significant. The expression of miR-146a in the experimental group was downregulated compared with that in the control group, as shown in **Figure 2F**.

#### 3.3.7 Analysis results of circulating miR-195-5p

Meta-analysis results of circulating miR-195-5p: I^2^ was 92.51%. The comprehensive SMD and 95% CI obtained by using the random effect model was 15.67 (9.60-21.74), P<0.05, which was statistically significant. The expression of miR-195-5p in the experimental group was upregulated compared with that in the control group, as shown in **Figure 2G**.

#### 3.3.8 Analysis results of circulating miR-451a

Meta-analysis results of circulating miR-451a: I^2^ was 97.43%. The comprehensive SMD and 95% CI obtained by using the random effect model was 10.83 (4.40~17.26), P<0.05, which was statistically significant. The expression of miR-451a in the experimental group was upregulated compared with that in the control group, as shown in **Figure 2H**.

#### 3.3.9 Analysis results of circulating miR-503

Meta-analysis results of circulating miR-503: I^2^ was 0.00%. The comprehensive SMD and 95% CI obtained by using a fixed effect model was 0.73 (0.27~1.19), P<0.05, which was statistically significant. The expression of miR-503 in the experimental group was upregulated compared with that in the control group, as shown in **Figure 2I**.

#### 3.3.10 Analysis results of circulating miR-708-5p

Meta-analysis results of circulating miR-708-5p: I^2^ was 93.85%. The random effect model was used to obtain its comprehensive SMD, and the 95% CI was -2.61 (-3.72~-1.50), P<0.05, which was statistically significant. The expression of miR-708-5p in the experimental group was downregulated compared with that in the control group, as shown in **Figure 2J**.

#### 3.3.11 Analysis results of circulating miR-146b-3p and miR-368

We did not perform a meta-analysis on circulating miR-146b-3p and miR-368 because only one publication on them was included in this study. According to the literature results, the SMD and 95% CI of circulating miR-146b-3p was -7.56 (-9.60~-5.53), indicating that the expression of miR-146b-3p in the experimental group was downregulated compared with that in the healthy control group. The SMD and 95% CI of circulating miR-368 was 4.07 (3.14~4.99), indicating that the expression of miR-368 in the experimental group was upregulated compared with that in the healthy control group.

### 3.4 Subgroup analysis

In this meta-analysis, the I^2^ values of circulating miR-223, miR-210, miR-200a, miR-126, miR-144, miR-146a, miR-195-5p, miR-451a, and miR-708-5p were all greater than 50%, showing great heterogeneity. Therefore, they need to be analyzed in subgroups. However, due to the lack of publications on circulating miR-210, miR-200a, miR-126 and miR-708-5p, subgroup analysis was not performed.

#### 3.4.1 Subgroup analysis of circulating miR-223

**Figure 3:**
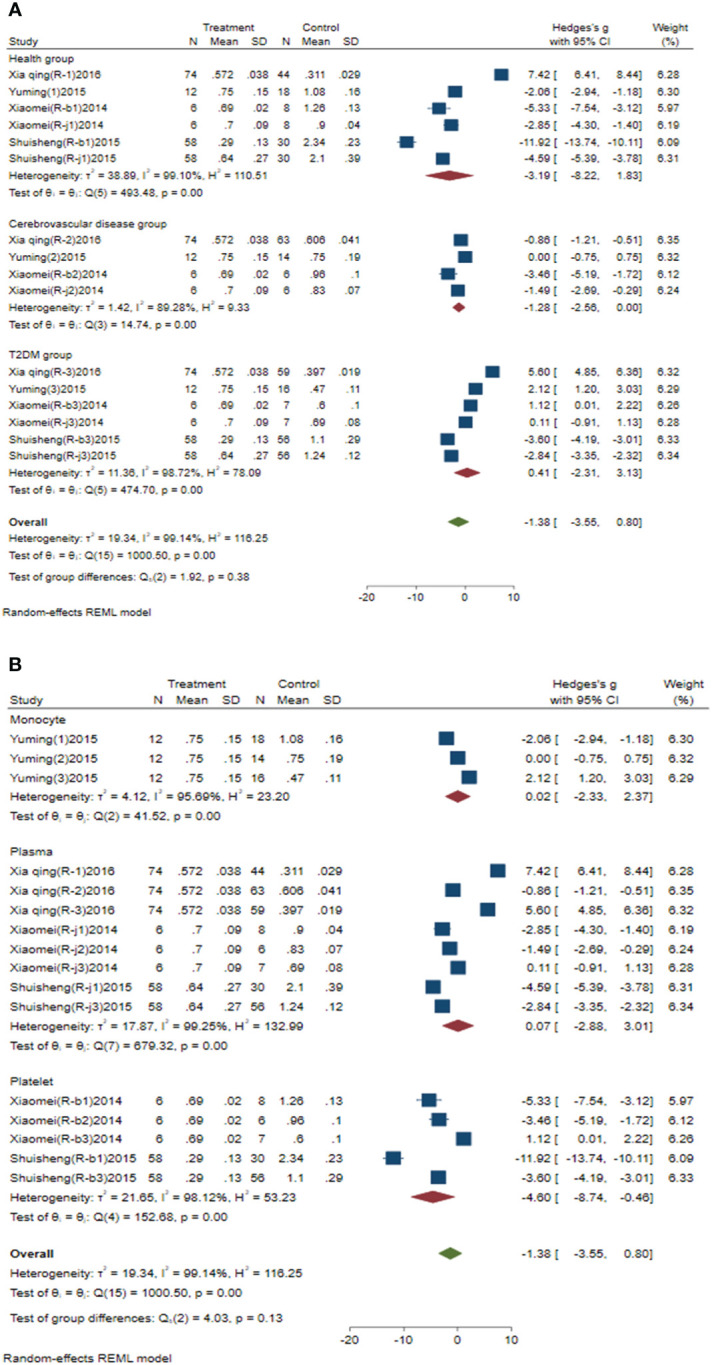
Forest plot of subgroup analysis of circulating miR-223 in type 2 diabetes mellitus with acute ischemic cerebrovascular disease. **(A)** Subgroup analysis of miR-223 by control group. **(B)** Subgroup analysis of miR-223 by sample source.

Subgroup analysis of circulating miR-223 was conducted according to the control group type and sample source. (1) According to the control group type (**Figure 3A**), the comprehensive SMD and 95% CI of circulating miR-223 expression in the study of the classification of the experimental group and healthy control group was -3.19 (-8.22-1.83), and I² was 99.10%. The comprehensive SMD and 95% CI expression in the study of the classification of the experimental group and the AICVD group was -1.28 (-2.56-0.00), and I² was 89.28%. The comprehensive SMD and 95% CI expression in the study of the classification of the experimental group and the T2DM group was 0.41 (-2.31~3.13), and I² was 98.72%. The direction of circulating miR-223 expression in the experimental group was inconsistent with that in the healthy group and the simple T2DM group, and it was downregulated compared with that in the simple AICVD group. (2) According to the sample source (**Figure 3B**), the comprehensive SMD and 95% CI of circulating miR-223 expression derived from monocytes was 0.02 (-2.33-2.37), and I² was 95.69%. In the studies derived from plasma, the comprehensive SMD and 95% CI was 0.07 (-2.88~3.01), and I² was 99.25%. In the studies derived from platelets, the comprehensive SMD and 95% CI was -4.60 (-8.74~-0.46), and I² was 98.12%. This indicates that the direction of circulating miR-223 expression in the experimental group is inconsistent with that in the control group in the studies derived from monocytes and plasma, and it is downregulated in the studies derived from platelets compared with that in the control group.

#### 3.4.2 Subgroup analysis of circulating miR-144

**Figure 4:**
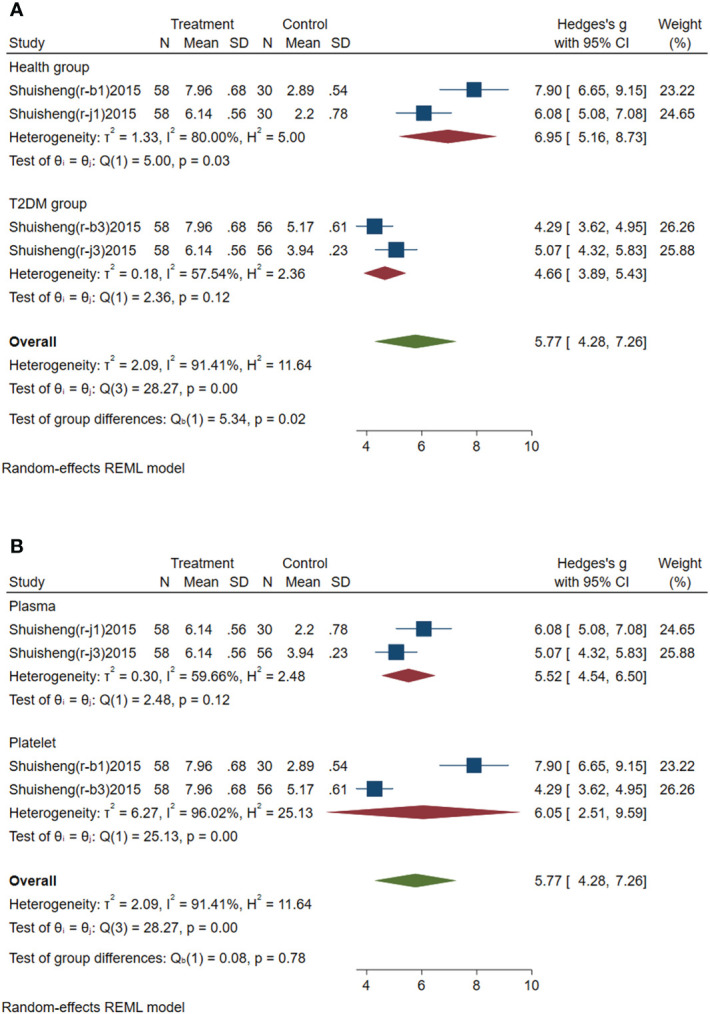
Forest plot of subgroup analysis of circulating miR-144 in type 2 diabetes mellitus with acute ischemic cerebrovascular disease. **(A)** Subgroup analysis of miR-144 by control group. **(B)** Subgroup analysis of miR-144 by sample source.

Subgroup analysis of circulating miR-144 was conducted according to the control group type and sample source. (1) According to the control group type (**Figure 4A**), the comprehensive SMD and 95% CI of circulating miR-144 expression in the study of the classification of the experimental group and healthy control group was 6.95 (5.16~8.73), and I² was 80.00%. The comprehensive SMD and 95% CI expression in the study of the classification of the experimental group and the T2DM group was 4.66 (3.89~5.43), and I² was 57.54%. The expression of circulating miR-144 in the experimental group was upregulated compared with that in the healthy group and simple T2DM group. (2) According to the sample source (**Figure 4B**), the comprehensive SMD and 95% CI of circulating miR-144 expression derived from plasma was 5.52 (4.54~6.50), and I² was 59.66%. In the studies derived from platelets, the comprehensive SMD and 95% CI was 6.05 (2.51~9.59), and I² was 96.02%. This indicated that the expression of circulating miR-144 in the experimental group was upregulated compared with that in the control group in the studies derived from both plasma and platelets.

#### 3.4.3 Subgroup analysis of circulating miR-146a

**Figure 5:**
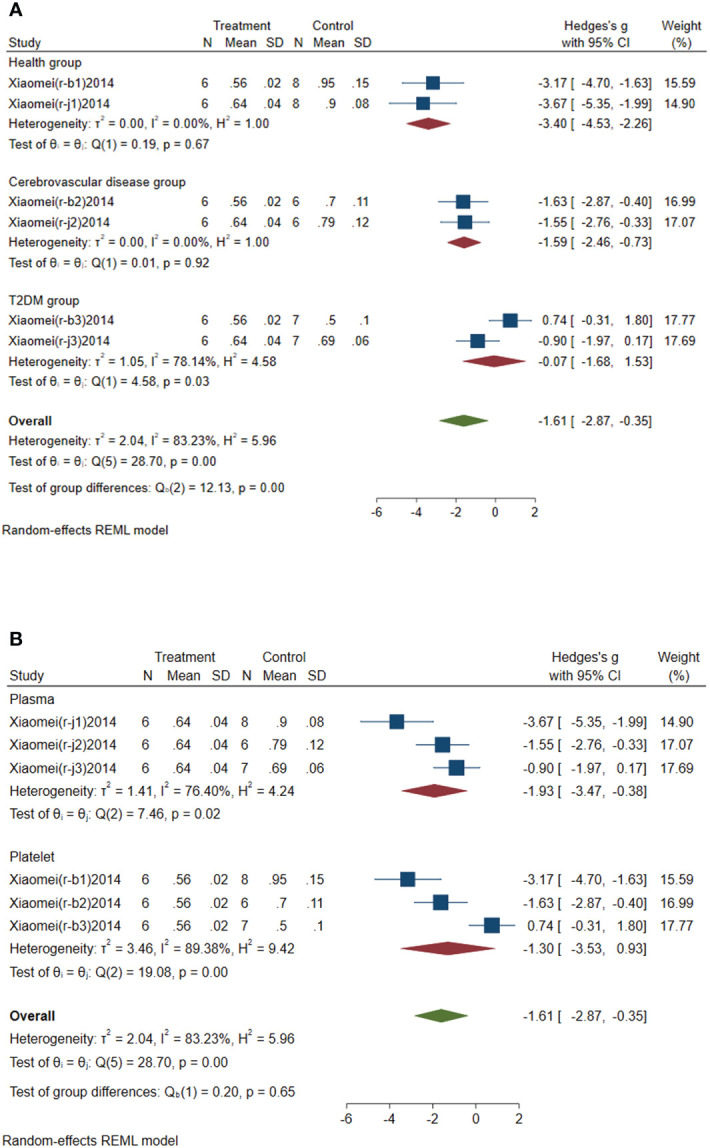
Forest plot of subgroup analysis of circulating miR-146a in type 2 diabetes mellitus with acute ischemic cerebrovascular disease. **(A)** Subgroup analysis of miR-146a by control group. **(B)** Subgroup analysis of miR-146a by sample source.

Subgroup analysis of circulating miR-146a was conducted according to the control group type and sample source. (1) According to the control group type (**Figure 5A**), the comprehensive SMD and 95% CI of circulating miR-146a expression in the study of the classification of experimental group and healthy group was -3.40 (-4.53~-2.26), and I² was 0.00%. The comprehensive SMD and 95% CI expression in the study of the classification of experimental group and the AICVD group was -1.59 (-2.46~-0.73), and I² was 0.00%. The comprehensive SMD and 95% CI expression in the study of the classification of experimental group and the T2DM group was -0.07 (-1.68~1.53), and I² was 78.14%. The direction of circulating miR-146a expression in the experimental group was downregulated compared with that in the healthy group and the simple AICVD group, and it was inconsistent compared with that in the simple T2DM group. (2) According to the sample source (**Figure 5B**), the comprehensive SMD and 95% CI of circulating miR-146a expression derived from plasma was -1.93 (-3.47~-0.38), and I² was 76.40%. In the studies derived from platelets, the comprehensive SMD and 95% CI was -1.30 (-3.53~0.93), and I² was 89.38%. This indicated that the direction of circulating miR-146a expression in the experimental group was downregulated compared with that in the control group in the studies derived from plasma, and it was inconsistent in the studies derived from platelets.

#### 3.4.4 Subgroup analysis of circulating miR-195-5p

**Figure 6:**
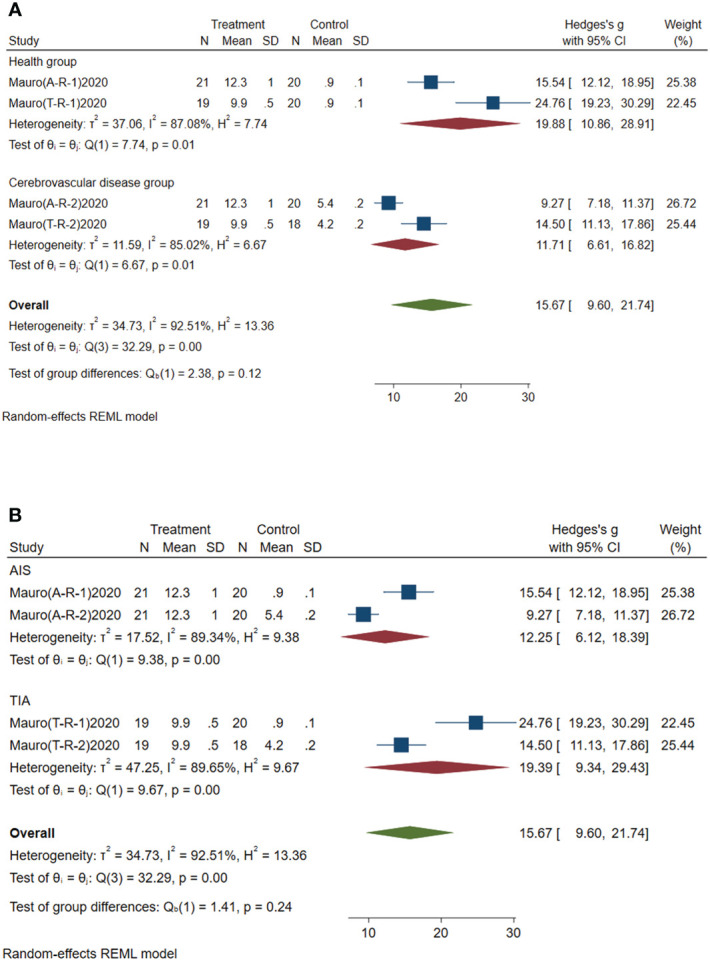
Forest plot of subgroup analysis of circulating miR-195-5p in type 2 diabetes mellitus with acute ischemic cerebrovascular disease. **(A)** Subgroup analysis of miR-195-5p by control group. **(B)** Subgroup analysis of miR-195-5p by disease type.

Subgroup analysis of circulating miR-195-5p was conducted according to the control group type and disease type. (1) According to the control group type (**Figure 6A**), the comprehensive SMD and 95% CI of circulating miR-195-5p expression in the study of the classification of the experimental group and healthy group was 19.88 (10.86~28.91), and I² was 87.08%. The comprehensive SMD and 95% CI expression in the study of the classification of the experimental group and the AICVD group was 11.71 (6.61~16.82), and I² was 85.02%. The expression of circulating miR-195-5p in the experimental group was upregulated compared with that in the healthy group and the simple AICVD group. (2) According to the disease type (**Figure 6B**), the comprehensive SMD and 95% CI of circulating miR-195-5p expression in the acute ischemic stroke (AIS) group was 12.25 (6.12~18.39), and I² was 89.34%. In the transient ischemic attack (TIA) group, the comprehensive SMD and 95% CI was 19.39 (9.34~29.43), and I² was 89.65%. This shows that the expression of circulating miR-195-5p in the experimental group was upregulated in the AIS and TIA groups compared with the control group.

#### 3.4.5 Subgroup analysis of circulating miR-451a

**Figure 7:**
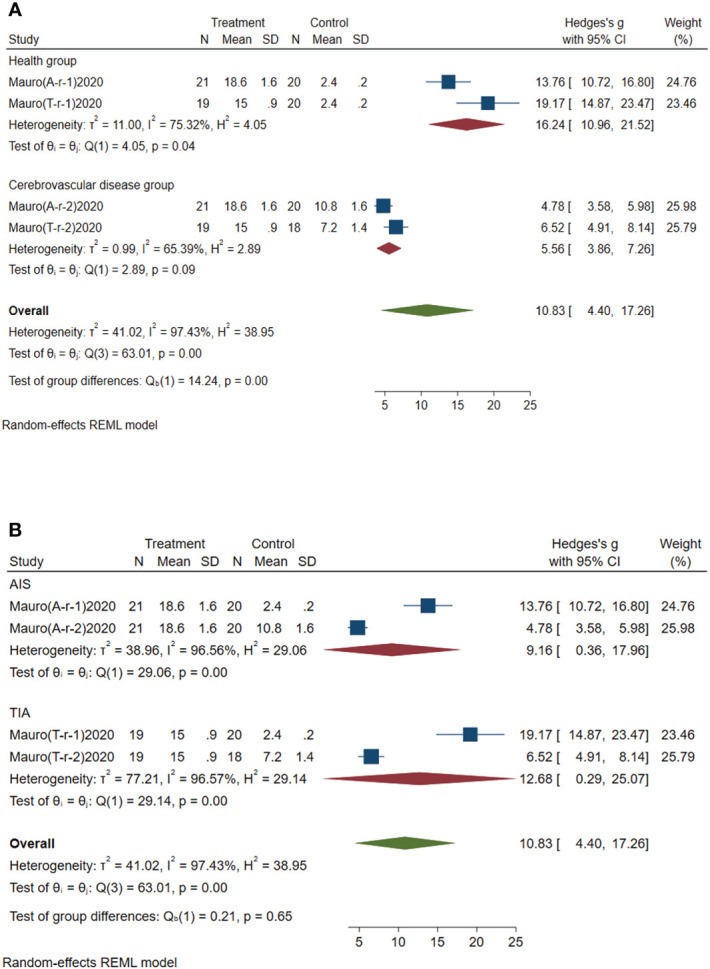
Forest plot of subgroup analysis of circulating miR-451a in type 2 diabetes mellitus with acute ischemic cerebrovascular disease. **(A)** Subgroup analysis of miR-451a by control group. **(B)** Subgroup analysis of miR-451a by disease type.

Subgroup analysis of circulating miR-451a was conducted according to the control group type and disease type. (1) According to the control group type (**Figure 7A**), the comprehensive SMD and 95% CI of circulating miR-451a expression in the study of the classification of the experimental group and healthy group was 16.24 (10.96~21.52), and I² was 75.32%. The comprehensive SMD and 95% CI expression in the study of the classification of the experimental group and the AICVD group was 5.56 (3.86~7.26), and I² was 65.39%. The expression of circulating miR-451a in the experimental group was upregulated compared with that in the healthy group and the simple AICVD group. (2) According to the disease type (**Figure 7B**), the comprehensive SMD and 95% CI of circulating miR-451a expression in the AIS group was 9.16 (0.36~17.96), and I² was 95.56%. In the TIA group, the comprehensive SMD and 95% CI was 12.68 (0.29~25.07), and I² was 96.57%. This shows that the expression of circulating miR-451a in the experimental group was upregulated in the AIS and TIA groups compared with the control group.

### 3.5 Meta regression analysis

The expression levels of circulating miR-223, miR-144, miR-146a, miR-195-5p, miR-200a, miR-210, miR-503 and miR-451a were analyzed by meta-regression. Analysis showed that the miRNA regulation direction (P=0.004<0.05) was the main source of miR-223 heterogeneity. The control group type (P=0.003<0.05) was the main source of miR-146a heterogeneity. The main sources of miR-195-5p heterogeneity were the control group type (P=0.000<0.05) and disease type (P=0.000<0.05). The control group type (P=0.009<0.05) was the main source of miR-200a heterogeneity. The miRNA regulatory direction (P=0.000<0.05) was the main source of miR-210 heterogeneity. The control group type (P=0.000<0.05) was the main source of miR-451a heterogeneity. The source of heterogeneity of miR-144 and miR-503 was not found.

### 3.6 Sensitivity analysis

**Figure 8:**
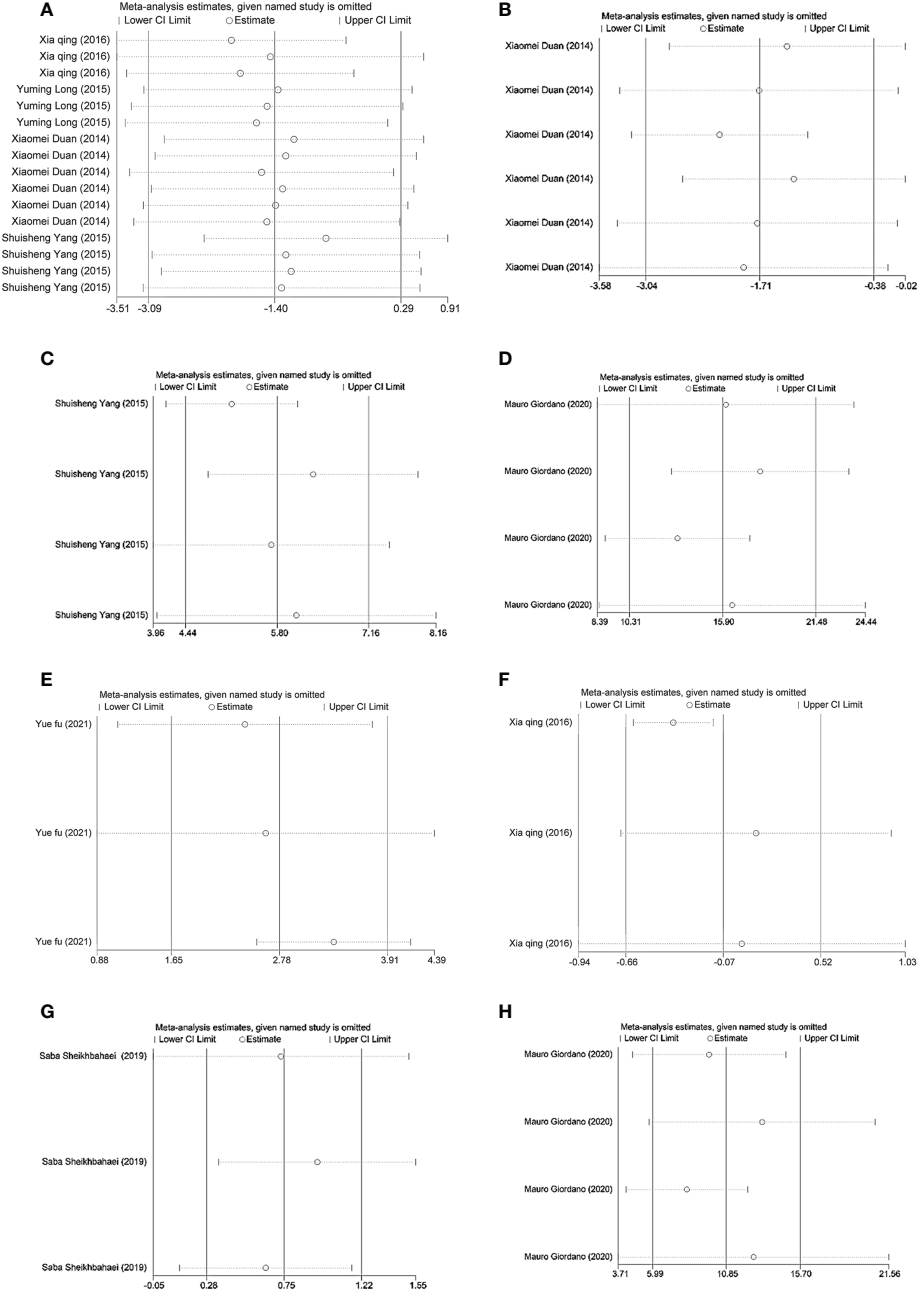
**(A)** Sensitivity analysis of miR-223. **(B)** Sensitivity analysis of miR-146a. **(C)** Sensitivity analysis of miR-144. **(D)** Sensitivity analysis of miR-195-5p. **(E)** Sensitivity analysis of miR-200a. **(F)** Sensitivity analysis of miR-210. **(G)** Sensitivity analysis of miR-503. **(H)** Sensitivity analysis of miR-451a.

Sensitivity analyses of miR-223 (**Figure 8A**), miR-146a (**Figure 8B**), miR-144 (**Figure 8C**), miR-195-5p (**Figure 8D**), miR-200a (**Figure 8E**), miR-210 (**Figure 8F**), miR-503 (**Figure 8G**) and miR-451a (**Figure 8H**) were performed. In all sensitivity analyses, we found that the results of the remaining studies did not change significantly after excluding each study separately, indicating that the sensitivity of the studies we included was low and that the results were robust and credible.

### 3.7 Publication bias test

**Figure 9:**
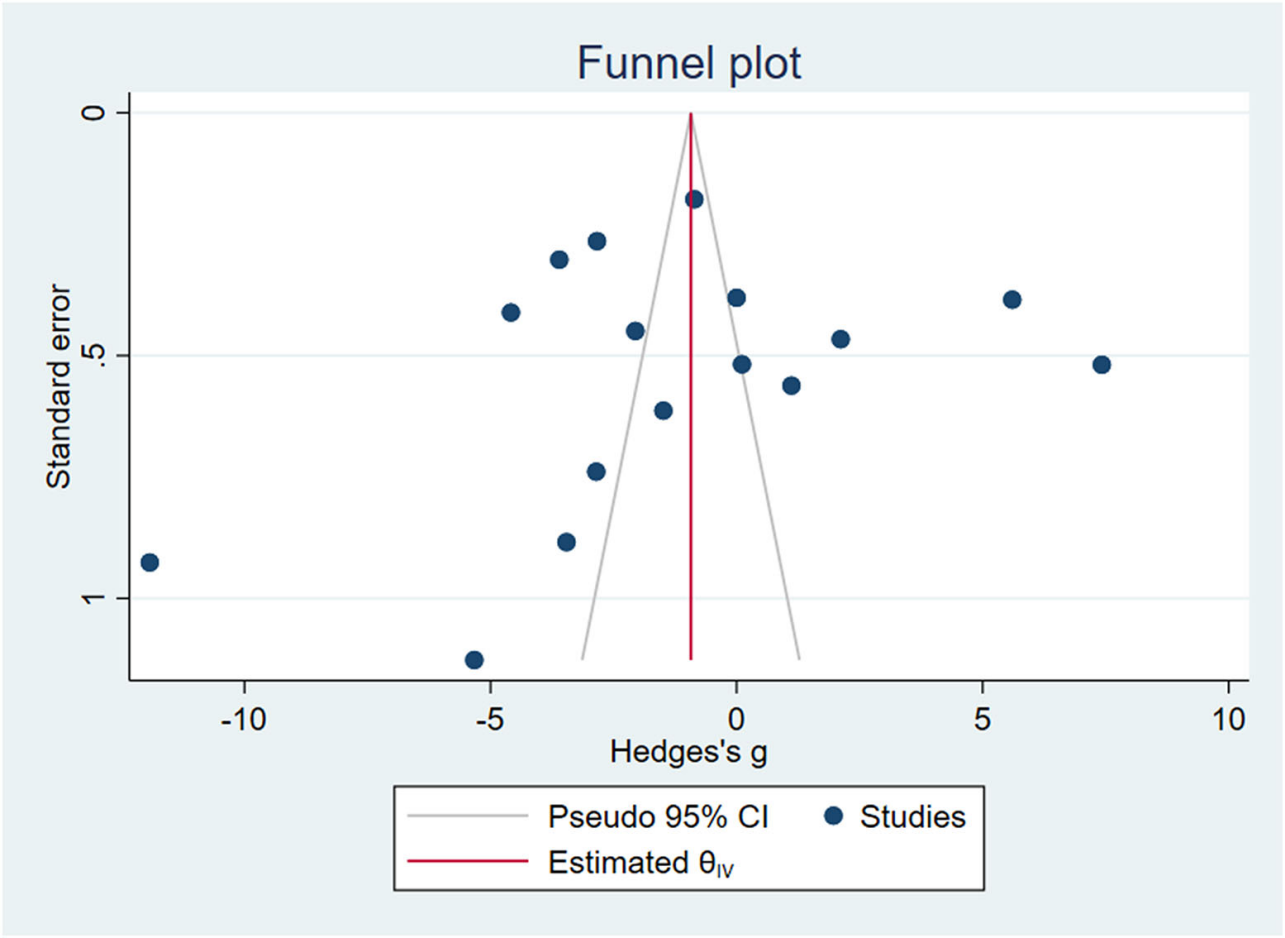
Funnel plot of circulating miR-223.

Stata16.0 was used to draw funnel plots. Due to the small number of other circulating miRNA studies, we only tested the publication bias of circulating miR-223, as shown in **Figure 9**, and obtained Egger’s test result of P=0.0604>0.05. We believe that there was publication bias.

## 4 Discussion

AICVD is a common complication of T2DM and has a high mortality rate. In recent years, the incidence rate has been increasing yearly. MRI and CTA, as the current diagnostic tools for T2DM with AICVD, can only be carried out in hospitals due to their complex operation, which largely limits the early identification of T2DM with AICVD in asymptomatic people. In addition, MRI examination has certain restrictions for patients, such as metal products in the body, claustrophobia and patients who cannot cooperate with the examination cannot perform MRI examination. CTA, on the other hand, has X-ray radiation and is not suitable for pregnant women and young children. Because the contrast agent is excreted by the kidneys, it may cause kidney damage and may worsen kidney function in patients with renal insufficiency.With a large number of studies in recent years, miRNAs have been found to play an important role in diabetes and its related macroscopic and microvascular complications (19). And due to their stable expression properties, miRNAs can be detected in various biological fluids, such as blood, saliva, urine or aqueous humor (20–22). Detection of circulating miRNAs by blood collection is a clear source because collection is minimally invasive and blood samples can be taken regularly at the clinic, so miRNAs show promise as a biomarker for the diagnosis, prognosis, and clinical monitoring of T2DM with AICVD.

This study mainly performed meta-analysis of the expression characteristics of miR-223, miR-210, miR-200a, miR-126, miR-144, miR-146a, miR-146b-3p, miR-195-5p, miR-368, miR-451a, miR-503 and miR-708-5p in blood samples in T2DM with AICVD.

Through analysis, it was found that the expression of serum miR-200a in the experimental group was upregulated compared with that in the healthy group, the simple AICVD group and the simple T2DM group. Yuefu Jiang (16) demonstrated that miR-200a could exert antioxidant effects on the arterial endothelium in T2DM at the gene level. In addition, WanW et al. (23) showed that miR-200a plays an important role in the development of DR (diabetic retinopathy) by affecting the viability, apoptosis and cell migration of HRMECs and may be a potential therapeutic target by downregulating PDLIM1 in DR. Therefore, we believe that circulating miR-200a may play a protective role in T2DM and its complications and can be used as a valuable molecular marker for the early identification of AICVD in T2DM.

The expression of miR-144 from plasma and platelets in the experimental group was upregulated compared with that in the healthy group and simple T2DM group. Shuisheng Yang et al. (17) showed in their study that the expression of miR-144 in the plasma and platelets of patients with type 2 diabetes mellitus was increased, which downregulated IRS-1 through the IRS-1-PI3K-Akt signaling pathway and then activated platelets, which increased the risk of ischemic stroke in patients with T2DM. Yu-Xiang Yan et al. (24) showed that miR-144 may play a role in the susceptibility of T2DM by changing the regulation of the stress response. In addition, Karolina et al. (25) demonstrated that peripheral blood miR-144 is a potential characteristic miRNA to distinguish IFG from T2DM. In conclusion, circulating miR-144 plays a role in the pathogenesis of T2DM and T2DM combined with AICVD and has specific expression. Therefore, we believe that it has potential diagnostic value in the early identification of T2DM combined with AICVD.

The expression of serum miR-503 was upregulated in the experimental group compared with the simple AICVD group, the simple T2DM group and the healthy group. Other studies have shown that miR-503 may inhibit the formation of neovascularization after diabetic ischemia. In addition, neutralizing miR-503 activity can also improve vascular healing and blood perfusion in ischemic limbs (26, 27). It has been demonstrated that miR-503 has an antiangiogenic effect in patients with T2DM and that miR-503 may increase the risk of stroke and disability in patients with diabetes mellitus by increasing inflammation and oxidative stress. Saba Sheikhbahaii (9) also showed that the differential expression of miR-503 among groups only occurred in the acute stage of ischemic cerebrovascular disease, and there was no significant difference in the expression of miR-503 among groups after 3 months. Therefore, miR-503 may be a potential diagnostic marker for AICVD in T2DM.

The expression of serum miR-126 in the experimental group was downregulated compared with that in the healthy group and simple T2DM group. Previous studies have shown that miR-126 is one of the most consistent miRs in diabetes (28, 29) and that it is mainly expressed in endothelial cells and plays a critical role in regulating endothelial cell function, angiogenesis, and vascular integrity (30, 31). Venkat et al. (32) concluded from mouse experiments that both T2DM and stroke significantly reduced the expression of miR-126, and the greatest reduction was observed in T2DM stroke mice. Venkat et al. also found that treatment with endothelial exosomes (EC-EXOs) in T2DM stroke mice significantly increased the expression of miR-126 in the serum and brain, improved neurological function and cognitive outcomes, and played an important role in the neurological recovery of T2DM stroke mice. Chen et al. (33) found in their study that T2DM significantly reduced the expression of miR-126 in the serum and brain tissue of T2DM mice. It was also demonstrated for the first time that human umbilical cord blood cell (HUCBC) treatment of stroke in T2DM mice increases serum, brain tissue, and brain endothelial cell miR-126 expression, thereby significantly decreasing BBB leakage and brain hemorrhage and increasing tight junction protein expression, promoting vascular and white matter remodeling, having antineuroinflammatory effects and improving functional outcomes after stroke. Based on the above studies on the diagnostic and therapeutic effects of miR-126 in T2DM mice with ischemic cerebrovascular disease, we speculate that circulating miR-126 has great value in the early identification and treatment of T2DM patients with ischemic cerebrovascular disease. Therefore, more relevant clinical studies can be carried out to confirm the diagnostic and therapeutic value of miR-126 in T2DM patients with AICVD.

In this study, the direction of circulating miR-223, miR-210 and miR-146a expression in the experimental group was inconsistent compared with that in the simple T2DM group, and there were still few related studies. Therefore, it is not considered a molecular marker for the early identification of T2DM complicated with AICVD. Alternatively, a large number of studies are still needed to explore its expression in T2DM complicated with AICVD.

In the included studies, the expression of circulating miR-195-5p and circulating miR-451a was upregulated in patients with T2DM complicated with AIS compared with healthy controls and patients with AIS alone. The expression of circulating miR-195-5p and circulating miR-451a was upregulated in patients with T2DM complicated with TIA compared with healthy controls and patients with TIA alone. The expression of circulating miR-708-5p in the experimental group was downregulated compared with that in the healthy group and the simple AICVD group. The expression of circulating miR-146b-3p in the experimental group was downregulated compared with that in the healthy group. The expression of circulating miR-368 in the experimental group was upregulated compared with that in the healthy group. However, there was no study on the differential expression of the above circulating miRNAs between the experimental group and the simple T2DM group in the literature we included. Therefore, it is not clear whether circulating miR-195-5p, miR-451a, miR-708-5p, miR-146b-3p and miR-368 can be used as valuable molecular markers for T2DM complicated with AICVD.

In this study, we analyzed and summarized the expression profile of miRNAs in T2DM with AICVD by synthesizing the existing findings. However, before translating miRNA profiles into clinical practice, studies using large, careful phenotypic cohorts and more technical standardization are necessary to identify miRNAs as effective and feasible markers for routine diagnosis and prognostic evaluation of T2DM with AICVD. On the one hand, there are few clinical studies on the specific expression of circulating miRNAs in T2DM with AICVD, and most of them are cross-sectional studies, and these results need to be further confirmed in large cohort studies whether they can be used as effective surrogates for current diagnostic tools. On the other hand, Baseline parameters, such as assessment of intra- and inter-individual variability, standardization of methods for all steps of sample collection and processing, RNA extraction, miRNA quantification, and analysis, will also need to be established to minimize interlaboratory biases. Vasu S et al. (34) pointed out that in different studies, the selection of endogenous miRNA controls may affect the results. In addition, such differences in small RNA input for cDNA conversion may introduce bias in the analysis. Small RNA sequencing avoids this bias by normalizing the expression to number of reads instead of the sample input. However, as small RNA sequencing is too expensive to perform on a routine basis. Absolute quantification of miRNA concentrations using synthetic miRNA mimics can help overcome these problems. Absolute quantification will also help in comparing different data sets and in correlation analysis. In summary, operational procedures should be standardized across studies for clinical use. We also found that miRNAs play a role in the pathogenesis of T2DM with AICVD. Improved understanding of the complex mechanisms underlying miRNAs dysregulation, and more well‐designed studies utilizing prospective samples would facilitate the translation of these miRNAs to clinical treatment methods.

In addition, in our included studies, the detection of circulating miRNAs in the experimental group (T2DM with AICVD group) was performed after AICVD was diagnosed by brain MRI in patients with T2DM, and we could only speculate on the basis of the current findings that they were also specifically expressed in T2DM patients who were about to develop AICVD. However, more clinical trials are needed to explore its value in assessing the risk of AICVD in patients with T2DM. For patients with abnormal expression of circulating miRNAs, further brain MRI is required to determine whether AICVD has occurred, and individualized intervention measures are taken in time to reduce the incidence and disability rate of AICVD.

In conclusion, we found that the expression of serum miR-200a, miR-503, plasma and platelet miR-144 was upregulated and the expression of serum miR-126 was downregulated in patients with T2DM complicated with AICVD. This may have diagnostic value in the early identification of T2DM complicated with AICVD. In addition, circulating miRNAs may play a role in the development of AICVD in patients with T2DM. More *in vivo* and *in vitro* studies are needed to further explore their roles and mechanisms to provide sufficient evidence-based medical evidence for clinical intervention of the disease.

## Data availability statement

The original contributions presented in the study are included in the article/**Supplementary Material**. Further inquiries can be directed to the corresponding authors.

## Author contributions

FH and LL wrote the manuscript and designed the study. XZ and GL analyzed the data. MC and ZL reviewed and edited the manuscript. All authors contributed to the article and approved the submitted version.
