# Supplementary material for: Meta-analysis of the characteristic expression of circulating microRNA in type 2 diabetes mellitus with acute ischemic cerebrovascular disease
Source: Front Endocrinol (Lausanne). 2023 Feb 14;14:1129860. doi: 10.3389/fendo.2023.1129860 (PMC9971585; doi:10.3389/fendo.2023.1129860)
Supplement: Supplementary file 1 [file Table_1.docx]

| **Table S1 Characteristics of included studies** | | | | | | | | |
| --- | --- | --- | --- | --- | --- | --- | --- | --- |
| The first author | Year | Country | Source | MiRNA expression profile | Assay type | Cases |  | Controls |
|  |  |  |  |  |  | Size Mean SD |  | Size Mean SD |
| Fan Cai[8] | 2018 | China | Monocyte | miR-146b-3p(Down) | RT‒qPCR | 15 0.16 0.07 |  | 15 1.12 0.16 |
| Saba Sheikhbahaei(2)[9] | 2019 | Iran | Serum | miR-503(Up) | RT‒qPCR | 15 3.29 2 |  | 18 1.88 1.4 |
| Saba Sheikhbahaei(3)[9] | 2019 | Iran | Serum | miR-503(Up) | RT‒qPCR | 15 3.29 2 |  | 12 2.49 1.8 |
| Saba Sheikhbahaei(1)[9] | 2019 | Iran | Serum | miR-503(Up) | RT‒qPCR | 15 3.29 2 |  | 5 1.1 0.4 |
| Xiaqing Guo(R-1)[10] | 2016 | China | Plasma | miR-223(Up) | RT‒qPCR | 74 0.572 0.038 |  | 44 0.311 0.029 |
| Xiaqing Guo(R-2)[10] | 2016 | China | Plasma | miR-223(Down) | RT‒qPCR | 74 0.572 0.038 |  | 63 0.606 0.041 |
| Xiaqing Guo(R-3)[10] | 2016 | China | Plasma | miR-223(Up) | RT‒qPCR | 74 0.572 0.038 |  | 59 0.397 0.019 |
| Xiaqing Guo(r-1)[10] | 2016 | China | Plasma | miR-210(Up) | RT‒qPCR | 74 1.21 0.29 |  | 44 1.01 0.46 |
| Xiaqing Guo(r-2)[10] | 2016 | China | Plasma | miR-210(Down) | RT‒qPCR | 74 1.21 0.29 |  | 63 1.37 0.41 |
| Xiaqing Guo(r-3)[10] | 2016 | China | Plasma | miR-210(Down) | RT‒qPCR | 74 1.21 0.29 |  | 59 1.28 0.18 |
| Zhen Liu(1)[11] | 2020 | China | Serum | miR-126(Down) | RT‒qPCR | 132 0.29 0.06 |  | 80 1.09 0.27 |
| Zhen Liu(3)[11] | 2020 | China | Serum | miR-126(Down) | RT‒qPCR | 132 0.29 0.06 |  | 74 0.68 0.23 |
| Dila Na(2)[12] | 2021 | China | Monocyte | miR-708-5p(Down) | RT‒qPCR | 92 0.96 0.17 |  | 100 1.48 0.31 |
| Dila Na(1)[12] | 2021 | China | Monocyte | miR-708-5p(Down) | RT‒qPCR | 92 0.96 0.17 |  | 100 2.26 0.54 |
| Yuming Long(1)[13] | 2015 | China | Monocyte | miR-223(Down) | RT‒qPCR | 12 0.75 0.15 |  | 18 1.08 0.16 |
| Yuming Long(2)[13] | 2015 | China | Monocyte | miR-223 | RT‒qPCR | 12 0.75 0.15 |  | 14 0.75 0.19 |
| Yuming Long(3)[13] | 2015 | China | Monocyte | miR-223(Up) | RT‒qPCR | 12 0.75 0.15 |  | 16 0.47 0.11 |
| Kaishun Meng(1)[14] | 2019 | China | Monocyte | miR-368(Up) | RT‒qPCR | 27 1.54 0.16 |  | 27 0.88 0.16 |
| Xiaomei Duan(R-b1)[15] | 2014 | China | Platelet | miR-223(Down) | RT‒qPCR | 6 0.69 0.02 |  | 8 1.26 0.13 |

| **Table S1 Characteristics of included studies (continued)** | | | | | | | | |
| --- | --- | --- | --- | --- | --- | --- | --- | --- |
| The first author | Year | Country | Source | MiRNA expression profile | Assay type | Cases |  | Controls |
|  |  |  |  |  |  | Size Mean SD |  | Size Mean SD |
| Xiaomei Duan(R-b2)[15] | 2014 | China | Platelet | miR-223(Down) | RT‒qPCR | 6 0.69 0.02 |  | 6 0.96 0.1 |
| Xiaomei Duan(R-b3)[15] | 2014 | China | Platelet | miR-223(Up) | RT‒qPCR | 6 0.69 0.02 |  | 7 0.6 0.1 |
| Xiaomei Duan(R-j1)[15] | 2014 | China | Plasma | miR-223(Down) | RT‒qPCR | 6 0.7 0.09 |  | 8 0.9 0.04 |
| Xiaomei Duan(R-j2)[15] | 2014 | China | Plasma | miR-223(Down) | RT‒qPCR | 6 0.7 0.09 |  | 6 0.83 0.07 |
| Xiaomei Duan(R-j3)[15] | 2014 | China | Plasma | miR-223(Up) | RT‒qPCR | 6 0.7 0.09 |  | 7 0.69 0.08 |
| Xiaomei Duan(r-b1)[15] | 2014 | China | Platelet | miR-146a(Down) | RT‒qPCR | 6 0.56 0.02 |  | 8 0.95 0.15 |
| Xiaomei Duan(r-b2)[15] | 2014 | China | Platelet | miR-146a(Down) | RT‒qPCR | 6 0.56 0.02 |  | 6 0.7 0.11 |
| Xiaomei Duan(r-b3)[15] | 2014 | China | Platelet | miR-146a(Up) | RT‒qPCR | 6 0.56 0.02 |  | 7 0.5 0.1 |
| Xiaomei Duan(r-j1)[15] | 2014 | China | Plasma | miR-146a(Down) | RT‒qPCR | 6 0.64 0.04 |  | 8 0.9 0.08 |
| Xiaomei Duan(r-j2)[15] | 2014 | China | Plasma | miR-146a(Down) | RT‒qPCR | 6 0.64 0.04 |  | 6 0.79 0.12 |
| Xiaomei Duan(r-j3)[15] | 2014 | China | Plasma | miR-146a(Down) | RT‒qPCR | 6 0.64 0.04 |  | 7 0.69 0.06 |
| Yuefu Jiang(1)[16] | 2021 | China | Serum | miR-200a(Up) | RT‒qPCR | 15 1.066 0.311 |  | 15 0.24 0.098 |
| Yuefu Jiang(2)[16] | 2021 | China | Serum | miR-200a(Up) | RT‒qPCR | 15 1.066 0.311 |  | 15 0.36 0.065 |
| Yuefu Jiang(3)[16] | 2021 | China | Serum | miR-200a(Up) | RT‒qPCR | 15 1.066 0.311 |  | 15 0.622 0.164 |
| Shuisheng Yang(r-b1)[17] | 2015 | China | Platelet | miR-144(Up) | RT‒qPCR | 58 7.96 0.68 |  | 30 2.89 0.54 |
| Shuisheng Yang(r-b3)[17] | 2015 | China | Platelet | miR-144(Up) | RT‒qPCR | 58 7.96 0.68 |  | 56 5.17 0.61 |
| Shuisheng Yang(r-j1)[17] | 2015 | China | Plasma | miR-144(Up) | RT‒qPCR | 58 6.14 0.56 |  | 30 2.2 0.78 |
| Shuisheng Yang(r-j3)[17] | 2015 | China | Plasma | miR-144(Up) | RT‒qPCR | 58 6.14 0.56 |  | 56 3.94 0.23 |
| Shuisheng Yang(R-b1)[17] | 2015 | China | Platelet | miR-223(Down) | RT‒qPCR | 58 0.29 0.13 |  | 30 2.34 0.23 |

| **Table S1 Characteristics of included studies (continued)** | | | | | | | | |
| --- | --- | --- | --- | --- | --- | --- | --- | --- |
| The first author | Year | Country | Source | MiRNA expression profile | Assay type | Cases |  | Controls |
|  |  |  |  |  |  | Size Mean SD |  | Size Mean SD |
| Shuisheng Yang(R-b3)[17] | 2015 | China | Platelet | miR-223(Down) | RT‒qPCR | 58 0.29 0.13 |  | 56 1.1 0.29 |
| Shuisheng Yang(R-j1)[17] | 2015 | China | Plasma | miR-223(Down) | RT‒qPCR | 58 0.64 0.27 |  | 30 2.1 0.39 |
| Shuisheng Yang(R-j3)[17] | 2015 | China | Plasma | miR-223(Down) | RT‒qPCR | 58 0.64 0.27 |  | 56 1.24 0.12 |
| Mauro Giordano(A-R-1)[18] | 2020 | Italy | Serum | miR-195-5p(Up) | RT‒qPCR | 21 12.3 1.0 |  | 20 0.9 0.1 |
| Mauro Giordano(A-R-2)[18] | 2020 | Italy | Serum | miR-195-5p(Up) | RT‒qPCR | 21 12.3 1.0 |  | 20 5.4 0.2 |
| Mauro Giordano(A-r-1)[18] | 2020 | Italy | Serum | miR-451a(Up) | RT‒qPCR | 21 18.6 1.6 |  | 20 2.4 0.2 |
| Mauro Giordano(A-r-2)[18] | 2020 | Italy | Serum | miR-451a(Up) | RT‒qPCR | 21 18.6 1.6 |  | 20 10.8 1.6 |
| Mauro Giordano(T-R-1)[18] | 2020 | Italy | Serum | miR-195-5p(Up) | RT‒qPCR | 19 9.9 0.5 |  | 20 0.9 0.1 |
| Mauro Giordano(T-R-2)[18] | 2020 | Italy | Serum | miR-195-5p(Up) | RT‒qPCR | 19 9.9 0.5 |  | 18 4.2 0.2 |
| Mauro Giordano(T-r-1)[18] | 2020 | Italy | Serum | miR-451a(Up) | RT‒qPCR | 19 15.0 0.9 |  | 20 2.4 0.2 |
| Mauro Giordano(T-r-2)[18] | 2020 | Italy | Serum | miR-451a(Up) | RT‒qPCR | 19 15.0 0.9 |  | 18 7.2 1.4 |

**Footnotes:** 1: The control group was healthy. 2: The control group was a simple acute ischemic cerebrovascular disease group. 3: The control group was a simple T2DM group.

J: Plasma source, b: Platelet source; A: AIS, T: TIA; R and r are used to distinguish different microRNAs in the same publication.
